# Analysis of Known Bacterial Protein Vaccine Antigens Reveals Biased Physical Properties and Amino Acid Composition

**DOI:** 10.1002/cfg.319

**Published:** 2003-10

**Authors:** Carl Mayers, Melanie Duffield, Sonya Rowe, Julie Miller, Bryan Lingard, Sarah Hayward, Richard W. Titball

**Affiliations:** Dstl Porton Down Salisbury Wiltshire SP4 0JQ United Kingdom

## Abstract

Many vaccines have been developed from live attenuated forms of bacterial pathogens or from killed bacterial cells. However, an increased awareness of the potential for transient side-effects following vaccination has prompted an increased emphasis on the use of sub-unit vaccines, rather than those based on whole bacterial cells. The identification of vaccine sub-units is often a lengthy process and bioinformatics approaches have recently been used to identify candidate protein vaccine antigens. Such methods ultimately offer the promise of a more rapid advance towards preclinical studies with vaccines. We have compared the properties of known bacterial vaccine antigens against randomly selected proteins and identified differences in the make-up of these two groups. A computer algorithm that exploits these differences allows the identification of potential vaccine antigen candidates from pathogenic bacteria on the basis of their amino acid composition, a property inherently associated with sub-cellular location.


**Comparative and Functional Genomics**
*Comp Funct Genom* 2003; **4**: 468–478.
Published online in Wiley InterScience (www.interscience.wiley.com). **DOI:** 10.1002/cfg.319

**Primary Research Paper**

### Analysis of known bacterial protein vaccine antigens reveals biased physical properties and amino acid composition

Carl Mayers, Melanie Duffield*, Sonya Rowe, Julie Miller, Bryan Lingard, Sarah Hayward and Richard W. Titball
*Dstl, Porton Down, Salisbury, Wiltshire SP4 0JQ, UK*

**Correspondence to:*
*Melanie Duffield, Dstl, Porton Down, Salisbury, Wiltshire SP4 0JQ, UK.*
*E-mail: mlduffield@dstl.gov.uk*

Received: 18 March 2003
Revised: 24 July 2003
Accepted: 28 July 2003

### Abstract

**Many vaccines have been developed from live attenuated forms of bacterial pathogens or from killed bacterial cells. However, an increased awareness of the potential for transient side-effects following vaccination has prompted an increased emphasis on the use of sub-unit vaccines, rather than those based on whole bacterial cells. The identification of vaccine sub-units is often a lengthy process and bioinformatics approaches have recently been used to identify candidate protein vaccine antigens. Such methods ultimately offer the promise of a more rapid advance towards pre-clinical studies with vaccines. We have compared the properties of known bacterial vaccine antigens against randomly selected proteins and identified differences in the make-up of these two groups. A computer algorithm that exploits these differences allows the identification of potential vaccine antigen candidates from pathogenic bacteria on the basis of their amino acid composition, a property inherently associated with sub-cellular location. Copyright © 2003 John Wiley & Sons, Ltd.**

**Keywords:** vaccine design; genome sequence; vaccine antigens

### Introduction

During the past 200 years the use of vaccines to control infectious diseases caused by bacterial pathogens has generally proved to be both effective and safe (Poland, 1999; Wilson and Marcuse, 2001). Many of these vaccines were discovered using an empirical approach (Nilsson, 2002) and included live attenuated forms of bacterial pathogens, killed bacterial cells and individual components of the bacterium (sub-units). Although many bacterial vaccines are still widely used, a shift towards reliance on antibiotics for the control of infectious diseases occurred during the latter half of the twentieth century.

The recent appearance of antibiotic resistant strains of many bacterial pathogens (Gould, 2002; Russell, 2002) has prompted a resurgence of interest in the use of vaccines to prevent disease. However, some vaccines are not considered to offer appropriate levels of protection against infection and there are still many infectious diseases for which effective vaccines are not available (Plotkin, 2001; Poland *et al.*, 2002). In addition, an increased awareness of the potential for transient or longer-term side-effects following vaccination (Plotkin, 2001; Wilson and Marcuse, 2001) has prompted an emphasis on the use of sub-unit vaccines.

Whilst empirical approaches to the selection of vaccine sub-units are still employed, bioinformatics approaches to select candidate protein sub-units from bacterial genome sequences have been used more recently (De Groot *et al.*, 2001; Gomez *et al.*, 2000; Montgomery, 2000; Nilsson, 2002; Pizza *et al.*, 2000; Ross *et al.*, 2001; Smith, 1996; Wizemann *et al.*, 2001). These approaches can be used to screen genomes for potential candidates far more rapidly than empirical approaches and have

Copyright © 2003 John Wiley & Sons, Ltd.

been termed 'reverse vaccinology' (Gomez *et al.*, 2000; Rappuoli, 2001).

Generally '*in silico*' approaches to the identification of vaccine antigens have relied on the assumption that candidate proteins will be located on the outer surface of, or exported from, the bacterium. Amino acid composition has been shown to be useful in the prediction of the sub-cellular location of proteins (Feng, 2002). Some workers have identified open reading frames (ORFs) that encode proteins possessing a signal sequence and screened this dataset to exclude proteins with transmembrane domains (Gomez *et al.*, 2000; Pizza *et al.*, 2000), and to include proteins with lipoprotein attachment sites (Chakravarti *et al.*, 2000; Gomez *et al.*, 2000) or other motifs associated with cell surface anchoring (Pizza *et al.*, 2000; Ross *et al.*, 2001). Other programs have been used to predict epitopes that bind to T cell receptors or major histocompatibility complexes to assist in vaccine design and development (Bond *et al.*, 2001; Grandi, 2001; Mallios, 1999, 2001; Savoie *et al.*, 1999). Whilst these various approaches have yielded novel sub-unit vaccines, the predictive power of these methods may be limited by our knowledge of protein export and processing pathways in different bacterial species, by the assumption that vaccine antigens will be surface-located and by our limited knowledge of the molecular architecture of outer membrane proteins.

We have set out to investigate whether the biophysical properties of reported protein vaccine antigens are significantly different from a representative control protein dataset.

### Materials and methods

### Construction of vaccine antigen dataset

Bacterial vaccine antigens were identified by patent and literature searches. To qualify for inclusion, the candidate, whole or part of the protein or corresponding DNA must have been shown to induce a protective response in an appropriate animal model after immunization. The amino acid sequences of the vaccine antigens were obtained from publicly available sequence databases, primarily from the National Centre for Biotechnology Information (**http://www.ncbi.nlm.nih.gov**).

### Construction of control dataset

A control dataset was constructed that mirrored the vaccine antigen dataset with respect to the proportion of entries from each genus. For each entry in the vaccine antigen dataset we randomly selected 35 proteins from the proteome of a representative species from the same genus. Where possible, the same species from the vaccine antigen dataset was used for the control dataset. In cases where an appropriate genome sequence was available but had not been annotated, the proteome was predicted using Glimmer, a system for finding genes in microbial DNA (**http://www.tigr.org/software.glimmer**) (Delcher *et al.*, 1999). Where no completed genome sequence was available for any member of the genus represented in the vaccine antigen dataset, all of the known proteins from a chosen species were downloaded from the publicly available protein sequence database (NCBI) and 35 proteins were then randomly selected.

### Removal of signal sequences

Known signal sequences of vaccine antigens were removed from entries in the vaccine antigen dataset. Proteins without a reported signal sequence, and all proteins in the control datasets, were separated into Gram-negative and Gram-positive entries and analysed using SignalP (Nielsen *et al.*, 1997); (**http://www.cbs.dtu.dk/services/SignalP**). Predicted signal sequences were removed to create two further datasets on which all comparisons of the vaccine antigen and control datasets were done.

### Construction of sub-cellular location protein datasets

A search of the SWISSPROT database (Swiss Institute of Bioinformatics; **http://www.expasy.ch/sprot**; Bairoch and Apweiler, 2000) identified proteins with defined sub-cellular locations for each of the bacterial species used to construct the control dataset. No entries were available in SWISSPROT for *Corynebacterium diptheriae*, so *Corynebacterium glutamicum* proteins were used instead. Any entries where the sub-cellular location of the protein was listed as 'putative', 'by similarity' or 'suggested' were omitted from the datasets. Separate datasets were constructed for each sub-cellular location, producing cytoplasmic (736 proteins), inner membrane (265 proteins), periplasmic

Copyright © 2003 John Wiley & Sons, Ltd. 

(77 proteins), outer membrane (99 proteins) and secreted proteins (94 proteins).

### Analysis of physical properties of proteins in the control and vaccine antigen datasets

Predicted molecular weights and predicted isoelectric points (pI) of proteins were calculated. Each protein in the control and vaccine antigen datasets was scored for hydrophobicity (Kyte and Doolittle, 1982), flexibility (Bhaskaram and Ponnuswamy, 1988), bulkiness (Zimmermann *et al.*, 1968) and relative mutability (Dayhoff *et al.*, 1978). The statistical significance of any differences was calculated by the Mann–Whitney test (Mann and Whitney, 1947; Wilcoxon, 1945). For all analyses, a $p$ score of <0.05 was considered to be significant.

### Calculation of amino acid composition of control and vaccine antigen datasets

The percentage amino acid composition of every protein was calculated. Statistically significant differences in amino acid composition between the control and vaccine antigen datasets were calculated by the Mann–Whitney test (Mann and Whitney, 1947; Wilcoxon, 1945).

### Development of scoring algorithms

The amino acid composition of each dataset was calculated as described above and the statistically significant differences noted. A score table was then produced, based on these differences. Each amino acid score was calculated using the mean dataset scores, as follows:

$$\text{Amino acid score} = \frac{\left(\begin{array}{c}\text{\% Composition of}\\ \text{vaccine antigen}\\ \text{dataset}\end{array} - \begin{array}{c}\text{\% Composition}\\ \text{of control}\\ \text{dataset}\end{array}\right)}{\text{\% Composition of control dataset/10}}$$

Amino acids more frequently found in the vaccine antigen dataset compared against the control dataset received a positive score, while those depleted in the vaccine antigen dataset received a negative score. Those that showed no statistically significant difference between the two datasets scored 0.

The scoring scale devised from the above analysis was used to score proteins in the vaccine antigen and control datasets as follows:

$$\text{Protein score} = \frac{\Sigma\text{Amino acid scores}}{\begin{array}{c}\text{Number of amino acids}\\ \text{in the protein}\end{array}}$$

The vaccine antigen scoring scale was applied to proteins from the sub-cellular datasets and the predicted proteome of *Streptococcus pneumoniae* strain R6 (Hoskins *et al.*, 2001).

### Construction of histograms

The distributions of scores from dataset comparisons are represented as histograms. Proteins from each of the two datasets being compared (a query dataset and a control) were scored according to published scales (for hydrophobicity, flexibility, bulkiness and relative mutability) or using the scales generated from amino acid sequences, as described previously. The scores from the query and control datasets were then combined and ranked. The range of scores generated was divided into 25 equal parts (histogram bins) that were used to represent the $x$ axis of the histogram. The upper limit of each bin is used as the axis label. The $y$ axis shows the percentage of proteins from each dataset that lies within each range of scores.

### Results

### Composition of the vaccine antigen and control dataset

In total, 72 non-homologous vaccine antigens were identified, originating from 32 bacterial species in 23 genera (Table 1) with 26 originating from Gram-positive bacteria and 46 from Gram-negative bacteria (for the purposes of this study, mycobacteria were treated as Gram-positive bacteria). A control dataset of 2520 proteins was constructed by randomly selecting 35 proteins from each representative species for each entry in the vaccine antigen dataset (Table 2). The size of the control dataset was selected so that it was approximately the number of proteins encoded by a typical bacterial genome. These vaccine antigen and control datasets were used for all subsequent comparisons. Of the proteins in the vaccine antigen dataset, 52(72%) were identified as having signal

Copyright © 2003 John Wiley & Sons, Ltd. 

**Table 1.** Proteins used to construct the vaccine antigen dataset

| Species | Antigen | Accession No. |
|---|---|---|
| *Bacillus anthracis* | Protective antigen (PA) | P13432 |
| *Bordetella pertussis* | *Pertussis* toxin S1 sub-unit | CAB51543 |
| *Bordetella pertussis* | Filamentous haemagglutinin (FHA) | S21010 |
| *Bordetella pertussis* | Pertactin (P69) | CAB40080 |
| *Borrelia burgdorferi* | Outer surface protein A (OspA) | S71533 |
| *Borrelia burgdorferi* | Outer surface protein B (OspB) | CAA32580 |
| *Borrelia burgdorferi* | Outer surface protein C (OspC) | S70290 |
| *Borrelia burgdorferi* | Virulent strain-associated repetitive antigen A (VraA) | NP_045547 |
| *Borrelia burgdorferi* | Outer membrane porin protein (Oms66/p66) | CAA61034 |
| *Borrelia burgdorferi* | Decorin binding protein A (DbpA) | AAD05353 |
| *Brucella abortus* | Cu/Zn superoxide dismutase | A33893 |
| *Brucella abortus* | 50S Ribosomal protein L7/L12 | P41106 |
| *Brucella melitensis* | Outer membrane protein 25(Omp25) | AAB06701 |
| *Campylobacter jejuni* | Flagellin (FlaA) | AAF05902 |
| *Chlamydia trachomatis* | Major outer membrane protein (MOMP) | P23732 |
| *Clostridium difficile* | Toxin A | P16154 |
| *Clostridium perfringens* | $\alpha$-Toxin (phospholipase C) | AAF20094 |
| *Clostridium perfringens* | $\varepsilon$-Toxoid (type D) | CAB60614 |
| *Clostridium tetani* | Tetanus toxin | AAF73267 |
| *Corynebacterium Pseudotuberculosis* | Phospholipase D | CAA01541 |
| *Escherichia coli* | Heat-labile enterotoxin (B sub-unit) | BAA25726 |
| *Escherichia coli* | Adhesin (FimH) | AAC77276 |
| *Haemophilus influenzae* | Fimbrin (P5) | P45996 |
| *Haemophilus influenzae* | Outer membrane protein P1 | AAF97552 |
| *Haemophilus influenzae* | Outer membrane protein P6 | P10324 |
| *Helicobacter pylori* | Cytotoxin-associated antigen(CagA) | AAD07614 |
| *Helicobacter pylori* | Heat shock protein 10 (Hsp10) | AAD07081 |
| *Helicobacter pylori* | Neutrophil-activating protein A (NapA) | AAF37843 |
| *Helicobacter pylori* | Citrate synthase (GltA) | AAD07097 |
| *Helicobacter pylori* | Urease (UreB) | BAA78630 |
| *Helicobacter pylori* | Vacuolating cytotoxin (VacA) | AAD07935 |
| *Helicobacter pylori* | Catalase | NP_223527 |
| *Legionella pneumophila* | Major secretory protein (MSP) | P21347 |
| *Legionella pneumophila* | Heat shock protein 60 (Hsp60/MCMP) | P26878 |
| *Legionella pneumophila* | Outer membrane protein S (OmpS) | A42596 |
| *Listeria monocytogenes* | Listeriolysin-O (LLO) | AAF64524 |
| *Listeria monocytogenes* | Major extracellular protein (P60) | P21171 |
| *Mycobacterium avium* | 65 kDa protein | AAA99670 |
| *Mycobacterium bovis* | MPB83 | Q10790 |
| *Mycobacterium bovis BCG* | Antigen 85A (Ag85A) | CAA37206 |
| *Mycobacterium bovis BCG* | Antigen 85B (Ag85B) | P12942 |
| *Mycobacterium tuberculosis* | Phosphate transport receptor PstS-3 (Ag88) | CAA88138 |
| *Mycobacterium tuberculosis* | Catalase-peroxidase (KatG) | CAB10056 |
| *Mycobacterium tuberculosis* | Antigen MPT63 | P97175 |
| *Mycobacterium tuberculosis* | Early secretory antigen target 6 (ESAT-6) | CAA17967 |
| *Neisseria meningitidis* | *Neisseria* surface protein A (NspA) | AAD53279 |
| *Neisseria meningitidis* | Transferrin binding protein (TbpA) | AAF81744 |
| *Pasteurella multocida* | *Pasteurella multocida* toxin (PMT) | P17452 |
| *Pseudomonas aeruginosa* | Outer membrane protein F (OprF) | AAG05166 |
| *Pseudomonas aeruginosa* | *Pseudomonas* exotoxin A (PEA) | AAB59097 |
| *Pseudomonas aeruginosa* | PcV | NP_250397 |
| *Rickettsia conorii* | Outer membrane protein A (OmpA) | Q52657 |
| *Rickettsia rickettsii* | Outer membrane protein B (OmpB) | Q53047 |
| *Rickettsia rickettsii* | Outer membrane protein A (OmpA) | P15921 |
| *Rickettsia tsutsugamushi* | MBP-Bor56 protein | AAA26375 |
| *Shigella dysenteriae* | *Shiga* toxin sub-unit B | P08027 |
| *Staphylococcus aureus* | Penicillin-binding protein (MecA) | BAB72132 |

Copyright © 2003 John Wiley & Sons, Ltd. 

**Table 1.** Continued

| Species | Antigen | Accession No. |
|---|---|---|
| *Staphylococcus aureus* | Fibrinogen binding protein | CAA79304 |
| *Staphylococcus aureus* | Collagen adhesin | A42404 |
| *Staphylococcus aureus* | Recomb SEA lacking superantigenic activity | P13163 |
| *Streptococcus agalactiae* | Surface immunogenic protein (Sip) | AAG18478 |
| *Streptococcus pneumoniae* | Pneumococcal surface protein A (PspA) | AAC62252 |
| *Streptococcus pneumoniae* | PhpA | AAK26629 |
| *Streptococcus pneumoniae* | Pneumolysin | A28568 |
| *Streptococcus pneumoniae* | Pneumococcal surface antigen A (PsaA) | AAF0668 |
| *Streptococcus pyogenes* | Fibronectin binding protein (Sfbl) | S54418 |
| *Treponema pallidum* | Glycerophosphodiester phosphodiesterase (Gpd) | AAB81591 |
| *Treponema pallidum* | Surface antigen 4D | P16665 |
| *Treponema pallidum* | TmpB antigen | F71283 |
| *Treponema pallidum* | TprK | AAF45141 |
| *Yersinia pestis* | F1 capsule antigen | CAA43966 |
| *Yersinia pestis* | V antigen | AAC62574 |

sequences. A lower proportion (253 of 2520; 14%) of proteins in the control dataset were predicted as having signal sequences.

### Physical properties of proteins in the vaccine antigen and control datasets

The isoelectric points (pI) and molecular weights were predicted for all proteins in the vaccine antigen and control datasets. The results were ranked and the distributions displayed as histograms (Figure 1a, b). The two-peak pattern of pI values seen with both the control and positive datasets was also seen with the predicted proteomes analysed from *Escherichia coli, Mycobacterium tuberculosis, Neisseria meningitidis* and *Streptococcus pneumoniae* (data not shown). The median values for each dataset were calculated and the Mann–Whitney test was applied. A comparison of positive and control datasets revealed statistically significant differences for both molecular weight and pI.

### Amino acid composition of vaccine antigen and control datasets

We analysed the amino acid compositions of the proteins in the vaccine antigen and control datasets using scales for hydrophobicity, flexibility, bulkiness or relative mutability, according to previously reported scoring methods (Bhaskaram and Ponnuswamy, 1988; Dayhoff *et al.*, 1978; Kyte and Doolittle, 1982; Zimmermann *et al.*, 1968). The output from each of these analyses was displayed

**Table 2.** Data sources of proteins used to construct the control dataset

| Genus | Data type and species | Data source |
|---|---|---|
| *Bacillus* | Proteome of *subtilis* | NCBI[1] |
| *Bordetella* | Genome of *pertussis* | Sanger Centre[2] |
| *Borrelia* | Proteome of *burgdorferi* | TIGR[3] |
| *Brucella* | Proteins from *melitensis* | NCBI |
| *Campylobacter* | Proteome of *jejuni* | Sanger Centre |
| *Chlamydia* | Proteome of *pneumoniae* | TIGR |
| *Clostridium* | Genome *acetobutylicum* | Genome Therapeutics[4] |
| *Corynebacterium* | Genome of *diptheriae* | Sanger Centre |
| *Escherichia* | Proteome of *coli* 0157 | University of Wisconsin[5] |
| *Haemophilus* | Proteome of *influenzae* | NCBI |
| *Helicobacter* | Proteome of *pylori* | TIGR |
| *Legionella* | Proteins from *pneumophila* | NCBI |
| *Listeria* | Proteome of *monocytogenes* | NCBI |
| *Mycobacterium* | Proteome of *tuberculosis* | Sanger Centre |
| *Neisseria* | Proteome of *meningitidis* | Sanger Centre |
| *Pasteurella* | Proteome of *multocida* | NCBI |
| *Pseudomonas* | Proteome of *aeruginosa* | NCBI |
| *Rickettsia* | Proteome of *prowazekii* | NCBI |
| *Shigella* | Proteins from *sonnei* | NCBI |
| *Staphylococcus* | Proteome of *aureus* | Sanger Centre |
| *Streptococcus* | Proteome of *pyogenes* | University of Oklahoma[6] |
| *Treponema* | Proteome of *pallidum* | TIGR |
| *Yersinia* | Proteome of *pestis* | Sanger Centre |

Proteins were selected from existing databases as shown: [1] **http://www.ncbi.nlm.nih.gov**; [2] **http://www.sanger.ac.uk**; [3] **http://www.tigr.org**; [4] **http://www.genomecorp.com**; [5] **http://www.genome.wisc.edu**; [6] **http://www.genome.ou.edu/**. Where a genome was used, the proteome was predicted using Glimmer. Where neither proteome or genome data was available, proteins for the selected species were randomly chosen from the NCBI protein database.

Copyright © 2003 John Wiley & Sons, Ltd. 

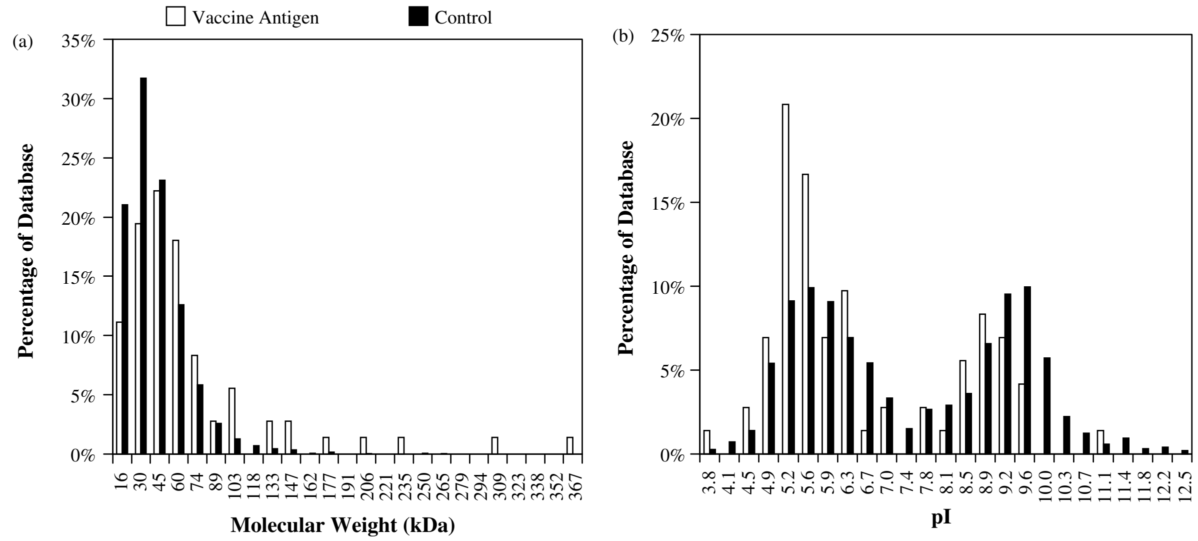

**Figure 1.** Vaccine antigen and control databases scored by predicted pI and molecular weight. Histograms are shown of the scores obtained by analysing the vaccine antigen and control databases for: (a) predicted molecular weight and (b) predicted pI. Histograms are constructed as described in Methods as a histogram (Figure 2a–d). The difference in the distribution of the scores from the positive and control datasets was statistically significant for each scale.

### Development of scoring algorithm

Although differences of the vaccine antigen and control datasets using the various published scales were statistically significant, the separation of distribution was poor, with a high percentage of one dataset falling within 1 SD of the mean of the other dataset (Table 3). We have devised a scoring system based on the average amino acid composition of all of the proteins in the positive and control datasets (Table 4). This scoring table was used to score individual proteins in the vaccine antigen and control datasets and the results of this analysis displayed as a histogram (Figure 3). A comparison of the positive and control datasets scored this way was statistically significant and a difference in the distribution of the scores was also seen with only around 18% of one dataset falling within 1 SD of the mean of the other dataset (Table 3).

### Vaccine scoring algorithm applied to other datasets

We considered that the differences in amino acid composition of the vaccine antigen and control datasets might reflect the differences in the likely cellular locations of the proteins. Therefore we applied the scoring algorithm to groups of proteins with known cellular locations (cytoplasmic, inner membrane, periplasmic, outer membrane or secreted) and compared each sub-cellular dataset against both the vaccine antigen and control datasets. There was no significant difference between the scores of known bacterial vaccine antigens and the scores of outer membrane or secreted

**Table 3.** Separation of distributions between vaccine antigen and control datasets using different scales

| | Proteins with scores within 1 SD of mean score of control dataset | | Proteins with scores within 1 SD of mean score of positive dataset | |
|---|---|---|---|---|
| Scale used | Control (%) | Positive (%) | Control (%) | Positive (%) |
| pI | 58.3 | 54.2 | 53.3 | 65.3 |
| Mol wt | 78.1 | 68.1 | 99.0 | 87.5 |
| Hydrophobicity | 75.8 | 73.6 | 57.0 | 73.6 |
| Mutability | 74.3 | 52.8 | 59.6 | 69.4 |
| Flexibility | 76.7 | 76.4 | 57.6 | 79.2 |
| Bulkiness | 75.6 | 54.2 | 38.2 | 68.1 |
| Our algorithm | 73.5 | 18.1 | 18.2 | 70.8 |

For each scale listed the percentage of scores in each dataset that falls within 1 SD of the mean of the control database or of the vaccine antigen database is given.

Copyright © 2003 John Wiley & Sons, Ltd. 

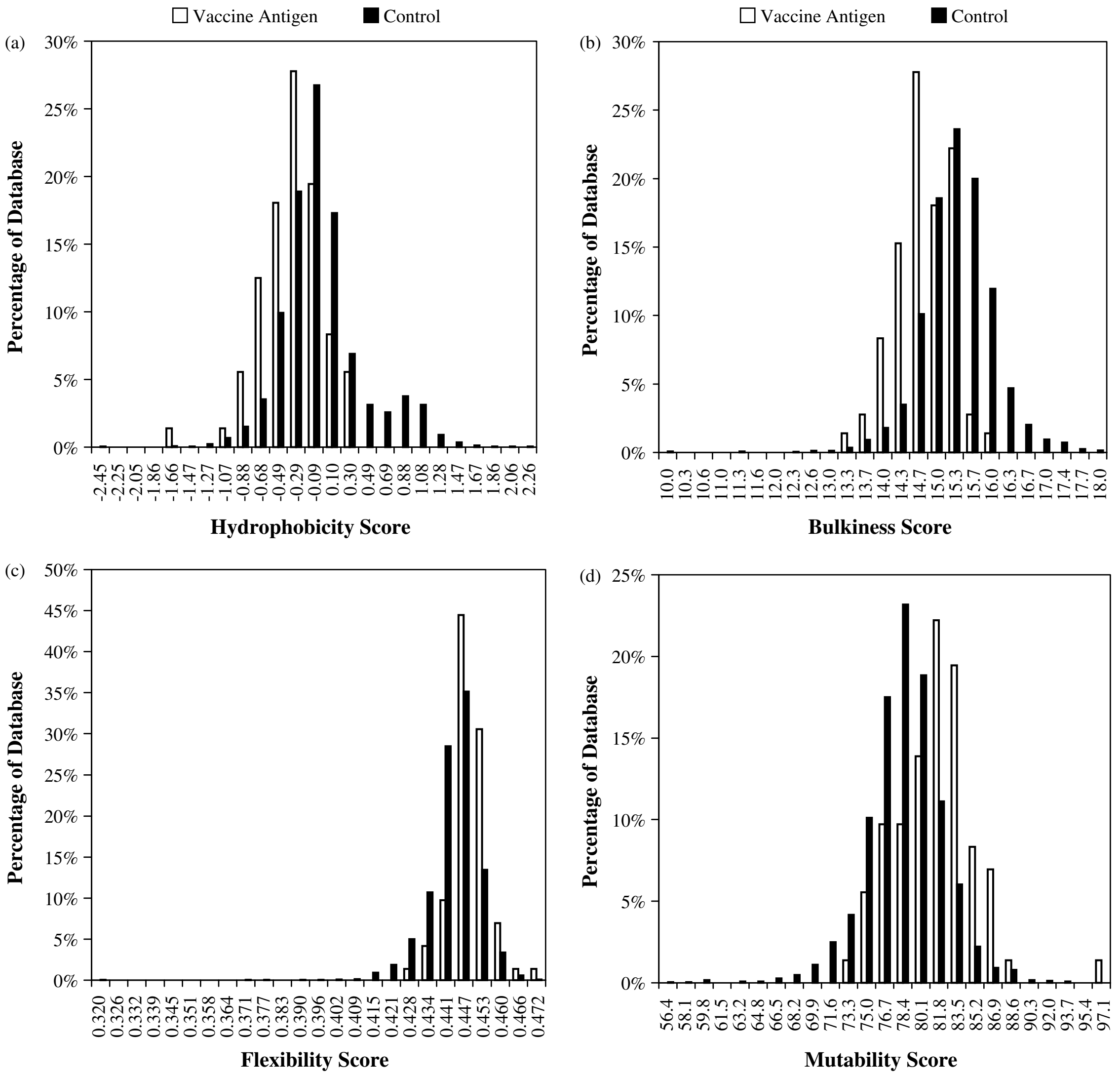

**Figure 2.** Vaccine antigen and control databases scored by four different scales. Histograms are shown of the scores obtained by scoring the vaccine antigen and control databases with: (a) Kyte–Doolittle hydrophobicity scale; (b) Zimmermann *et al.* bulkiness scale; (c) Bhaskaran and Ponnuswamy flexibility scale; and (d) Dayhoff *et al.* relative mutability scale. Histograms are constructed as described in Methods proteins Table 5. The control dataset showed no bias to any one sub-cellular location.

### Vaccine scoring algorithm applied to a test proteome

To evaluate the algorithm, we analysed the proteome of *S. pneumoniae* R6 (2043 proteins) and ranked the proteins by score. The vaccine antigen database contains four entries from *S. pneumoniae*. When ranked, pneumococcal surface protein A (PspA), was the highest ranked (10th) of these four known protective antigens, with the other three vaccine antigens ranking within the top 10% (within the first 204 proteins when ranked by score; Table 6). Potential vaccine candidates from *S. pneumoniae* N4 (Wizemann *et al.*, 2001), and known pneumococcal virulence factors that may also have potential as vaccine antigens (Jedrzejas, 2001) were also found within the top 10% of proteins when ranked by our scoring algorithm. Of the five proteins identified by Wizemann *et al.*,

Copyright © 2003 John Wiley & Sons, Ltd. 

**Table 4.** Amino acid composition of vaccine antigen and control databases

| Amino acid | Vaccine antigen Mean | Vaccine antigen SD | Control Mean | Control SD | *p* | Score |
|---|---|---|---|---|---|---|
| A | 9.39 | 4.13 | 8.41 | 4.21 | 0.039 | 1.17 |
| C | 0.60 | 0.81 | 1.12 | 1.23 | 0.000 | −4.64 |
| D | 6.17 | 2.19 | 5.21 | 2.20 | 0.000 | 1.84 |
| E | 6.22 | 3.53 | 6.05 | 2.80 | 0.385 | 0 |
| F | 3.17 | 1.52 | 4.39 | 2.56 | 0.000 | −2.78 |
| G | 8.36 | 3.21 | 6.93 | 3.13 | 0.000 | 2.06 |
| H | 1.63 | 1.47 | 2.14 | 1.51 | 0.000 | −2.38 |
| I | 5.08 | 2.02 | 7.16 | 3.41 | 0.000 | −2.91 |
| K | 7.48 | 3.92 | 6.41 | 3.87 | 0.040 | 1.67 |
| L | 7.46 | 2.20 | 10.06 | 3.29 | 0.000 | −2.58 |
| M | 1.60 | 1.13 | 2.43 | 1.32 | 0.000 | −3.42 |
| N | 6.24 | 2.64 | 4.50 | 2.61 | 0.000 | 3.87 |
| P | 3.68 | 2.04 | 3.83 | 2.08 | 0.295 | 0 |
| Q | 3.77 | 1.83 | 3.67 | 2.01 | 0.305 | 0 |
| R | 3.18 | 2.01 | 5.19 | 3.14 | 0.000 | −3.87 |
| S | 6.99 | 2.92 | 6.25 | 2.39 | 0.052 | 0 |
| T | 7.27 | 3.19 | 5.04 | 2.10 | 0.000 | 4.42 |
| V | 6.99 | 2.09 | 6.84 | 2.66 | 0.396 | 0 |
| W | 1.18 | 1.04 | 1.01 | 1.02 | 0.109 | 0 |
| Y | 3.79 | 2.02 | 3.33 | 1.95 | 0.061 | 0 |

The mean percentage amino acid composition and SD of the proteins within the vaccine antigen and control databases are listed. The probability (*p*) of the two databases sharing the same median has been calculated by the Wilcoxon Rank Sum test and is given to three decimal places. Values of *p* below 0.05 are significantly different and have been allocated a score, as indicated in Methods.

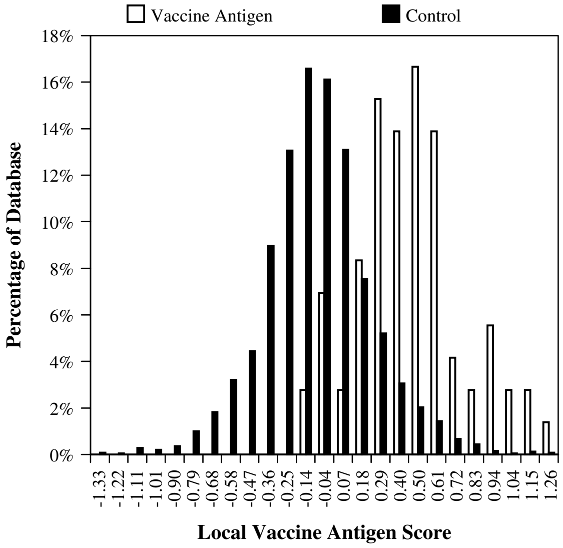

**Figure 3.** Vaccine antigen and control databases scored by vaccine antigen scale. A histogram is shown of the scores obtained by scoring the vaccine antigen and control databases with the vaccine antigen scale. The histogram constructed as described in Methods

**Table 5.** *p* Scores for comparisons of the vaccine antigen and control datasets against datasets of various sub-cellular locations

| Dataset | Probability of sharing the same median with: Positive dataset | Control dataset |
|---|---|---|
| Cytoplasmic | $6.8 \times 10^{-30}$ | $1.5 \times 10^{-10}$ |
| Inner membrane | $1.6 \times 10^{-25}$ | $1.6 \times 10^{-4}$ |
| Periplasmic | $2.0 \times 10^{-5}$ | $2.1 \times 10^{-21}$ |
| Outer membrane | 0.08 | $2.9 \times 10^{-38}$ |
| Secreted | 0.33 | $1.3 \times 10^{-35}$ |

The vaccine antigen scale was used to score proteins from either the vaccine antigen or control dataset and datasets of proteins from various cellular locations. The *p* score (the probability that two datasets share the same median) was calculated by the Wilcoxon Rank Sum test.

SP101, a conserved hypothetical protein with a signal peptidase II cleavage site motif, had the lowest ranking of all vaccines and potential vaccine antigens at 376 (Table 6).

Predicted signal sequences were removed from the *S. pneumoniae R6* proteome and ranked again as described above. Slight changes in rankings were observed; however, all but SP101 were again found to rank within the top 10% (Table 6). Of the top 100 pneumococcal proteins ranked by our algorithm, 31 were predicted to possess a signal sequence.

### Discussion

The genome sequences of many bacterial pathogens have now been determined and this has prompted significant work to investigate how these genome sequences can be interpreted to provide improved pre-treatments or therapies for disease. Previous workers have used a range of methods to identify vaccine antigens. Some workers have assumed that vaccine antigens are located on the surface of the bacterium, and used algorithms that predict the cellular location to interrogate the predicted bacterial proteome for novel vaccine candidates (Gomez *et al.*, 2000). Others have used algorithms to locate proteins with sequence homology to known vaccines (Adamou *et al.*, 2001; Moxon *et al.*, 2002). Such techniques would fail to predict new families of vaccine candidates. Other reported methods involve the identification of tandem repeats at the

Copyright © 2003 John Wiley & Sons, Ltd. 

**Table 6.** Proteins of *Streptococcus pneumoniae* R6 scored by the vaccine antigen scale

| *S. pneumoniae* R6 Protein | Rank SS | Score SS | Rank w/o SS | Score w/o SS |
|---|---|---|---|---|
| Choline-binding protein G (CBP) | 5 | 2.17 | 8 | 2.17 |
| Surface protein pspA precursor* (CBP)† | 10 | 1.96 | 12 | 2.04 |
| Choline-binding protein A (CBP)† | 17 | 1.83 | 14 | 1.93 |
| Choline-binding protein (CBP) | 27 | 1.72 | 36 | 1.72 |
| Choline-binding protein F (CBP)† | 34 | 1.65 | 19 | 1.88 |
| Autolysin (LytA) | 79 | 1.28 | 86 | 1.28 |
| ABC transporter protein–Mn transport PsaA* | 81 | 1.26 | 88 | 1.26 |
| Neuraminidase (NanB) | 90 | 1.23 | 102 | 1.23 |
| Hyaluronate lyase | 122 | 1.11 | 134 | 1.11 |
| Endo-$\beta$-N-acetylglucosaminidase (CBP)—Sp46 | 139 | 1.05 | 150 | 1.05 |
| Pneumolysin* | 152 | 0.99 | 168 | 0.99 |
| Pneumococcal histidine triad protein A r PhpA* | 170 | 0.93 | 181 | 0.93 |
| Neuraminidase (NanA) | 183 | 0.89 | 193 | 0.89 |
| Conserved hypothetical protein—Sp101 | 376 | 0.55 | 385 | 0.55 |

Known vaccine antigens and virulence factors of *Streptococcus pneumoniae* scored and ranked by the vaccine antigen scale are listed. Proteins that are included in the vaccine antigen database are denoted by*. Proteins with Sp numbers are vaccine antigen candidates, as identified by Weizman *et al.* (2001). CBP denotes a choline-binding protein. † Predicted to have a signal sequence. SS, proteome used inclusive of signal sequences; W/o SS, proteome used with predicted signal sequences removed.

5′ end of a gene, since such repeats have been associated with some virulence genes (Hood *et al.*, 1996). However, many virulence-associated genes lack such repeats and would not have been identified using this method. We have extended these approaches to identify the likely properties of vaccine antigens by comparing the amino acid composition of known protein vaccine antigens with those of randomly selected proteins in a control dataset.

It has been a generally held hypothesis that secreted or surface-located proteins are most likely to induce a protective immune response (Grandi, 2001). *In silico* methods have therefore been employed to identify potential vaccine antigens by predicting secreted proteins by searching for signal sequences (Chakravarti *et al.*, 2000; Gomez *et al.*, 2000; Janulczyk and Rasmussen, 2001). Our analysis has confirmed for the first time that a higher proportion of protein vaccine antigens have signal sequences when compared to the control dataset (72% vs. 14%).

Protein antigens having no classic leader sequence would fail to be identified using methods searching for signal sequences, such as ESAT-6 from *M. tuberculosis* (Li *et al.*, 1999; Olsen *et al.*, 2001; Sorensen *et al.*, 1995). Using our scoring algorithm, ESAT-6 was ranked 92nd out of the 3918 proteins in the entire predicted proteome of *M. tuberculosis* (i.e. in the top 3%).

The $p$ scores of both predicted pI and molecular weights of the proteins in the positive dataset showed statistically significant differences from the control dataset. The bimodal pattern of the pI values occurred with all of the datasets analysed and confirms previous observations with bacterial and archaeal proteomes (Van Bogelen *et al.*, 1999). Since proteins are generally less soluble around their isoelectric points, and the cytoplasm has a pH value near to neutrality, it has been suggested that cytoplasmic proteins rarely have a neutral pI.

Our analysis has revealed that the hydrophobicity, bulkiness, flexibility and mutability of vaccine antigens are significantly different from these properties of our control dataset. As most vaccine antigens previously described are surface-exposed or secreted, they are more likely to be in contact with surrounding media. This might be reflected in their hydrophobicity and may therefore explain the differences seen between the two datasets using hydrophobicity as a scale. The difference in mutability could reflect the ability of pathogens to alter their antigenic presentation and thereby evade the host's immune system. Phenotypic variation in the relevant cell-surface proteins has been seen amongst clinical isolates of some species, suggesting that antigenic proteins can mutate and evolve during the period of infection (Peterson *et al.*, 1995). This could also account for the differences seen in the comparisons of bulkiness, molecular weights and flexibility since the use of small, flexible residues on a protein surface may also reflect the capability to mutate. The difference in molecular weight reflects the size ranges of the two datasets. The control datasets ranges from 1.62 to 252 kDa, whilst the vaccine antigen dataset ranges from 7.69 to 367 kDa. The overlap between the two datasets does not allow this property to be used to predict vaccine antigen proteins. The greatest difference in separation of distribution between the vaccine antigen and control datasets

Copyright © 2003 John Wiley & Sons, Ltd.

was achieved when amino acid compositions were compared. The algorithm we derived exploits these differences.

Using *Streptococcus pneumoniae* R6 as a test proteome, our scoring algorithm was able to rank the known antigens included in our vaccine antigen dataset within the top 10% of *S. pneumoniae* proteins — other bacterial proteomes have also been ranked using our scoring algorithm, and the known vaccine antigens occur most frequently in the top 10% of scores (data not shown). Other virulence factors and potential vaccine candidates from *S. pneumoniae* were also ranked within the top 10% of scores.

This study demonstrates a fast and efficient scoring system that utilizes amino acid composition as a tool for the prediction of vaccine candidates. Construction of the vaccine antigen dataset has confirmed that a high proportion of known antigens have signal sequences. Since this scoring system is based on amino acid composition, secreted and outer membrane proteins score highly using the algorithm described. However, since this method does not rely on sequence similarity or motifs, it should also identify vaccine candidates lacking such features that other prediction tools, using these criteria, may miss. Ranking proteomes by this method has shown that known protective antigens score highly, independently of cellular location or possession of signal sequences. In contrast to previous methods, our algorithm uses data derived only from bacterial proteins and therefore is specific for use with bacterial genomes. This scoring system therefore provides a fast and efficient method of ranking whole bacterial proteomes for potential vaccine antigen candidates. We aim to use the datasets and algorithms to predict novel vaccine candidates from pathogenic bacteria that will form the basis for clinical trials.

### Acknowledgement

This work was supported by UK Ministry of Defence.

### References

Adamou J, Heinrichs J, Erwin A, *et al.* 2001. Identification and characterization of a novel family of pneumococcal proteins that are protective against sepsis. *Infect Immun* **69**: 949–958.

Bairoch A, Apweiler R. 2000. The SWISS-PROT protein sequence database and its supplement TrEMBL in 2000. *Nucleic Acids Res* **28**: 45–48.

Bhaskaram R, Ponnuswamy P. 1988. Positional flexibility of amino acid residues in globular proteins. *Int J Pept Protein Res* **32**: 242–255.

Bond K, Sriwanthana B, Hodge T, *et al.* 2001. An HLA-directed molecular and bioinformatics approach identifies new HLA-A11 HIV-1 subtype E cytotoxic T lymphocyte epitopes in HIV-1-infected *Thais*. *AIDS Res Human Retroviruses* **17**: 703–717.

Chakravarti D, Fiske M, Fletcher L, Zagursky R. 2000. Mining genomes and mapping proteomes: identification and characterization of protein subunit vaccines. *Dev Biol (Basel)* **103**: 81–90.

Dayhoff M, Schwartz R, Orcutt B. 1978. A model of evolutionary change in proteins. In *Atlas of Protein Sequence and Structure*, Dayhoff M (ed.). National Biomedical Research Foundation: Washington, DC; 345–352.

De Groot A, Bosma A, Chinai N, *et al.* 2001. From genome to vaccine: *in silico* predictions, *ex vivo* verification. *Vaccine* 4385–4395.

Delcher A, Harmon D, Kasif S, White O, Salzberg S. 1999. Improved microbial gene identification with Glimmer. *Nucleic Acids Res* **27**: 4636–4641.

Feng Z-P. 2002. An overview on predicting the subcellular location of a protein. *In Silico Biol* **2** [**http://www.bioinfo.de/isb/toc_vol_02.htm**]. Article 0027.

Gomez M, Johnson S, Gennaro M. 2000. Identification of secreted proteins of *Mycobacterium tuberculosis* by a bioinformatic approach. *Infect Immun* **66**: 2323–2327.

Gould I. 2002. Antibiotic policies and control of resistance. *Curr Opin Genet Dev* **15**: 398–400.

Grandi G. 2001. Antibacterial vaccine design using genomics and proteomics. *Trends Biotechnol* **19**: 181–188.

Hood D, Deadman M, Jennings M, *et al.* 1996. Use of whole genome sequence of *Haemophilus influenzae* to identify novel virulence genes associated with DNA repeats. *Proc Natl Acad Sci USA* **93**: 11 121–11 125.

Hoskins J, Alborn WJ, Arnold J, *et al.* 2001. Genome of the bacterium *Streptococcus pneumoniae* strain R6. *J Bacteriol* **183**: 5709–5717.

Janulczyk R, Rasmussen M. 2001. Improved pattern for genome-based screening identifies novel cell wall-attached proteins in Gram-positive bacteria. *Infect Immun* **69**: 4019–4026.

Jedrzejas M. 2001. Pneumococcal virulence factors: structure and function. *Microbiol Mol Biol Rev* **65**: 187–207.

Kyte J, Doolittle R. 1982. A simple method for displaying the hydropathic character of a protein. *J Mol Biol* **157**: 105–132.

Li Z, Howard A, Kelley C, Delogu G, Collins F, Morris S. 1999. Immunogenicity of DNA vaccines expressing tuberculosis proteins fused to tissue plasminogen activator signal sequences. *Infect Immun* **67**: 4780–4786.

Mallios R. 1999. Class II MHC quantitative binding motifs derived from a large molecular database with a versatile iterative stepwise discriminant analysis meta-algorithm. *Bioinformatics* **15**: 432–439.

Mallios R. 2001. Predicting class II MHC/peptide multi-level binding with an iterative stepwise discrimination analysis meta-algorithm. *Bioinformatics* **17**: 942–948.

Mann H, Whitney D. 1947. On a test whether one of two random variables is stochastically larger than the other. *Ann Math Statist* **18**: 50–60.

Montgomery D. 2000. Tuberculosis vaccine design: influence of the completed genome sequence. *Briefings Bioinformat* **1**: 289–296.

Copyright © 2003 John Wiley & Sons, Ltd. 

Moxon E, Hood D, Saunders N, Schweda E, Richards J. 2002. Functional genomics of pathogenic bacteria. *Phil Trans R Soc Lond B* **357**: 109–116.

Nielsen H, Engelbrecht J, Brunak S, von Heijne G. 1997. Identification of prokaryotic and eukaryotic signal peptides and prediction of their cleavage sites. *Protein Eng* **10**: 1–6.

Nilsson C. 2002. Bacterial proteomics and vaccine development. *Am J Pharmacogenom* **2**: 59–65.

Olsen A, van Pinxteren L, Okkels L, Rasmussen P, Andersen P. 2001. Protection of mice with a tuberculosis subunit vaccine based on a fusion protein of antigen 85B and ESAT-6. *Infect Immun* **69**: 2773–2778.

Peterson S, Bailey C, Jenson J, *et al*. 1995. Characterisation of repetitive DNA in the *Mycoplasma genitalium* genome: possible role in the generation of antigenic variation. *Proc Natl Acad Sci USA* **92**: 11 829–11 833.

Pizza M, Scarlato V, Masignani V, *et al*. 2000. Identification of vaccine candidates against serotype B meningococcus by whole-genome sequencing. *Science* **287**: 1816–1820.

Plotkin S. 2001. Lessons learned concerning vaccine safety. *Vaccine* **20**: S16–S19.

Plotkin S. 2001. Vaccines in the 21st century. *Infect Dis Clin North Am* **15**: 307–327.

Poland G. 1999. Current paradoxes and changing paradigms in vaccinology. *Vaccine* **17**: 1605–1611.

Poland G, Murray D, Bonilla-Guerrero R. 2002. New vaccine development. *Br Med J* **324**: 1315–1319.

Rappuoli R. 2001. Reverse vaccinology, a genome-based approach to vaccine development. *Vaccine* **19**: 2688–2691.

Ross B, Czajkowski L, Hocking D, *et al*. 2001. Identification of vaccine candidate antigens from a genomic analysis of *Porphyromonas gingivalis*. *Vaccine* **19**: 4135–4142.

Russell A. 2002. Antibiotic and biocide resistance in bacteria: introduction. *J Appl Microbiol* **92**: 1S–3S.

Savoie C, Kamikawaji N, Sasazuki T, Kuhara S. 1999. Use of BONSAI decision trees for the identification of potential MHC class I peptide epitope motifs. *Pac Symp Biocomput* **4**: 182–189.

Smith D. 1996. Microbial pathogen genomes — new strategies for identifying therapeutics and vaccine targets. *Trends Biotechnol* **14**: 290–293.

Sorensen A, Nagai S, Houren G, Anderson P, Anderson A. 1995. Purification and characterisation of a low-molecular-mass T-cell antigen secreted by *Mycobacterium tuberculosis*. *Infect Immun* **63**: 1710–1717.

Van Bogelen R, Schiller E, Thomas J, Neidhardt F. 1999. Diagnosis of cellular states of microbial organisms using proteomics. *Electrophoresis* **20**: 2149–2159.

Wilcoxon F. 1945. Individual comparisons by ranking methods. *Biometrics* **1**: 80–83.

Wilson C, Marcuse E. 2001. Vaccine safety–vaccine benefits: science and the public's perception. *Nature Rev Immunol* **1**: 160–165.

Wizemann T, Heinrichs J, Adamou J, *et al*. 2001. Use of a whole genome approach to identify vaccine molecules affording protection against *Streptococcus pneumoniae* infection. *Infect Immun* **69**: 1593–1598.

Zimmermann J, Eliezer N, Simha R. 1968. The characterization of amino acid sequences in proteins by statistical methods. *J Theoret Biol* **21**: 170–201.

Copyright © 2003 John Wiley & Sons, Ltd. 
